# Electroanalytical Overview: Electrochemical Sensing Platforms for Food and Drink Safety

**DOI:** 10.3390/bios11080291

**Published:** 2021-08-23

**Authors:** Alejandro Garcia-Miranda Ferrari, Robert D. Crapnell, Craig E. Banks

**Affiliations:** Faculty of Science and Engineering, Manchester Metropolitan University, Manchester M1 5GD, UK; A.Garcia-Miranda.Ferrari@mmu.ac.uk (A.G.-M.F.); R.Crapnell@mmu.ac.uk (R.D.C.)

**Keywords:** electroanalytical sensors, drug detection, bacteria detection, virus detection, allergen detection, food safety, drink safety, food and drink

## Abstract

Robust, reliable, and affordable analytical techniques are essential for screening and monitoring food and water safety from contaminants, pathogens, and allergens that might be harmful upon consumption. Recent advances in decentralised, miniaturised, and rapid tests for health and environmental monitoring can provide an alternative solution to the classic laboratory-based analytical techniques currently utilised. Electrochemical biosensors offer a promising option as portable sensing platforms to expedite the transition from laboratory benchtop to on-site analysis. A plethora of electroanalytical sensor platforms have been produced for the detection of small molecules, proteins, and microorganisms vital to ensuring food and drink safety. These utilise various recognition systems, from direct electrochemical redox processes to biological recognition elements such as antibodies, enzymes, and aptamers; however, further exploration needs to be carried out, with many systems requiring validation against standard benchtop laboratory-based techniques to offer increased confidence in the sensing platforms. This short review demonstrates that electroanalytical biosensors already offer a sensitive, fast, and low-cost sensor platform for food and drink safety monitoring. With continued research into the development of these sensors, increased confidence in the safety of food and drink products for manufacturers, policy makers, and end users will result.

## 1. Introduction

The World Health Organisation (WHO) states that sufficient access to safe and nutritious food is crucial to sustaining life and promoting good health. Foodborne diseases are usually caused by bacteria, viruses, parasites, or chemical compounds that enter the body from polluted food or water, causing harm to the human body. An estimated 10% of the world’s population fall ill after ingesting contaminated food, with around 420,000 people dying from it every year [1]. Foodborne diseases hinder socioeconomic development by increasing the burden on health care systems and harming tourism, trade, and economies, and can cause a vicious cycle of malnutrition and disease particularly among children, the elderly, and those more vulnerable [1].

Currently, food and water products are transported beyond national borders; therefore, well-established standards and effective governmental, producer, and consumer collaboration help ensure food safety standards are maintained. It is for that reason that the Codex Alimentarius (or “Food Code”) was produced. It is a collection of standards, guidelines, and codes of good practice proposed and adopted by the 188 members (i.e., countries) of the Codex Alimentarius Commission (CAC), which is a central part of the joint Food and Agriculture Organisation by the United Nations and the WHO (FAO-WHO) Food Standards Programme, which was established in 1963 to promote and protect fair practices and consumer health in the food trade [2]. To find out which Codex standards have been adopted or how can they be used, the latest copy of the List of Standards in reference [3] should be sought. Pathogens can enter the body from water or undercooked/contaminated food; therefore, it is of the highest priority to differentiate the presence of those pathogens before they enter the body [4,5].

Multiple analytical methods, such as gas chromatography (GC) [6], high-performance liquid chromatography (HPLC) [7], enzyme-linked immunosorbent assay (ELISA) [8], and lateral flow immunoassay [9] have been used for foodborne disease detection [10]. Traditional analytical methods include desktop-based equipment and culture-based, immunological-, nucleic- and biosensor-based detection methods which, although selective, often require complex sample and equipment preparation added to labour-intensive and time-consuming methods in some cases [11] and, therefore, not ideal for the large-scale manufacture of sensors towards on-site, decentralised, and affordable food safety analysis. Spectroscopic methods such as Raman, surface-enhanced Raman spectroscopy (SERS), infrared (IR), or ultraviolet (UV) spectroscopy also offer portable, rapid, sensitive, and non-destructive food safety monitoring sensors. Among these, Raman and SERS exhibit higher spectral resolution and narrower bandwidths, which translates into multi-elemental and multiplex detection [12,13,14]. Strong efforts are being made in order to couple SERS-based sensors with microfluidic modules and control interfaces together in handheld devices; however, they have not achieved yet such small sizes as electrochemical potentiostats and commercialised sensors. Some recent trends to enhance the capabilities of Raman and SERS sensors is the use of chemometric analysis to build databases and calibration curves/plots in order to distinguish small differences from a variety of complex sample matrices. Although the cost of these on-site spectroscopic sensors has decreased dramatically in recent years, more efforts are needed to offer truly affordable alternatives to electrochemical on-site sensors. Interested readers can find further information on Raman, SERS, and smartphone-based optical assays for food safety monitoring, within the following recent reviews [12,13,14].

**Figure 1:**
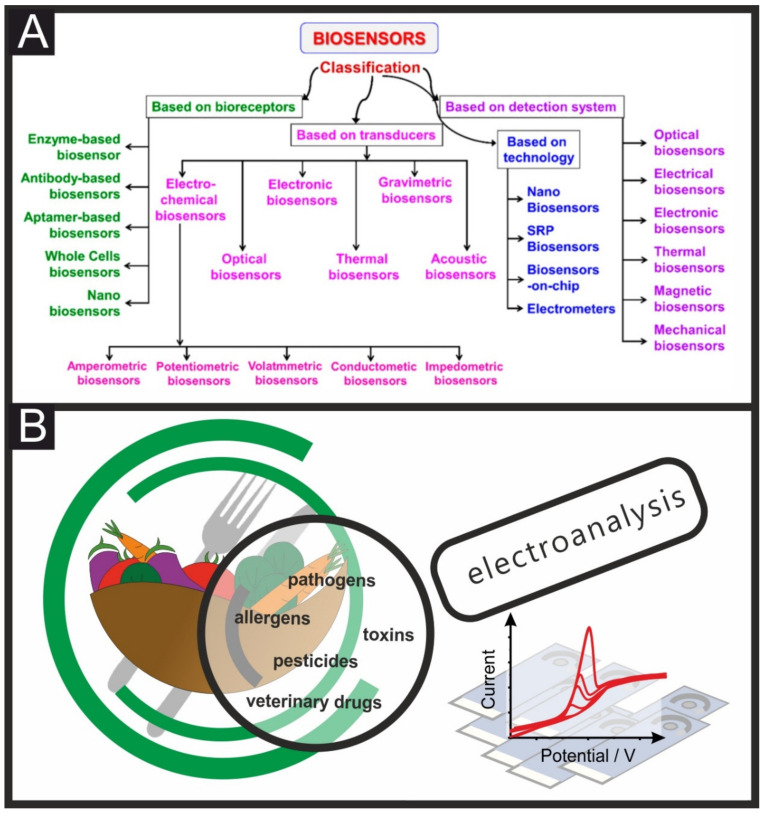
Classification of biosensors based on their bioreceptors, transducers, technology or detection system (**A**). Schematic representation of some of the multiple applications of tailored electrochemical sensing platforms towards food safety (**B**).

Indeed, modern electroanalytical methods offer a rapid, robust, and powerful analytical solution with miniaturised, bulk-manufactured, and affordable sensors, with little or no sample preparation whilst being a suitable solution for environmental [15,16,17], forensics [18,19,20] biomedical [21,22] and food [23,24] control applications, to name just a few [25]; Electrochemical methods achieve high selectivity and sensitivity when careful attention is paid to tailor the electrode material, recognition elements, electrochemical technique, potentials, etc. to the target analyte [26,27]. Multiple examples of successful food safety biosensors are presented in the literature, see Table 1; however, many of them utilise time-consuming electrode preparation that would be difficult to scale up when commercialisation occurs; herein, therefore, we want to highlight the importance of bulk modification and manufacture of biosensors when developing these type of on-site analysis devices. Biosensors can be classified depending on their recognition element and/or their transduction signalling. Figure 1 shows a biosensor classification (A) and schematic of the importance of electroanalysis in food and drink quality control, including a variety of target analytes that can be detected (B). As shown in Figure 1A, one of the main components of biosensors is their bioreceptors, which can be enzymatic (most common), immunosensors (highly specific and sensitive), aptamers or nucleic acid-based, or microbial or whole-cell biosensors [28]. A classification based on the transducer and sensors can be also performed, dividing the sensors as follows: electrochemical, electronic, thermal, optical, or gravimetric sensors. Other categorisations can be performed such as analyte–bioreceptor combination, detection systems, and type of applied technologies [28].

## 2. Electrochemical Sensors towards Food Safety

The presence of undeclared substances in food, especially when processed, is a complicated challenge for monitoring agencies due to the complex manufacturing, processing, handling, etc., especially when heating and fermentation steps are applied [72]. Recent scientific discoveries and analytical methods have allowed us to increase our understanding of living organisms and their/our metabolism, which has helped identify a variety of bioreceptors from biological organisms [73]. The discovery of these bioreceptors has helped introduce a new generation of electrochemical biosensors that can provide an alternative to classical analytical methods for the trace detection of food safety analytes. The most common determination methods for food safety can be categorised between immunological and DNA-based assays, or between direct and indirect methods (depending on whether the analyte in question is the harmful molecule itself or a characteristic biomarker of its presence) [74]. Although these methods provide excellent results and are well understood by researchers in the field, complete validation against the mentioned lab-based methodologies is required to garner end-user confidence from people not within the field.

Immunological methods use the specific recognition involved in the antigen-antibody coupling; apta- and genosensors use DNA/RNA to bind to the particular analyte or to detect the encoding genes of the particular analyte via hybridisation with complementary DNA sequences [73]. Imprinted polymers (IPs), or plastic antibodies, are also used as a recognition scaffold to create exclusive non-covalent binding sites to the target analyte, be it a small molecule [75], protein [76,77], or microorganism [78,79,80]. Affinity assay methods, similar to ELISA tests, either sandwich or competitive, are also applied towards food safety. Label-free non-competitive methods are also applied by immobilising the bioreceptor directly on the transducer’s surface. In terms of electrochemical methods, these are often using the following procedures: amperometry, potentiometry, impedance, and voltammetry. Although progress is still needed in order to expand and have mass-acceptance for the use of electrochemical biosensors in food safety monitoring, in this review, we compile and explain some elegant and recent literature examples of novel electrochemical biosensors towards food safety. We divide these into the different target molecules—namely, pathogens, toxins, allergens, viruses, veterinary drugs, forensic drugs, and pesticides. For an in-depth look into the recent advances in the portable sensing of heavy metals, we refer the reader to a recent review paper [17]. Due to the importance of these areas and the plethora of work undertaken in these fields, we will predominantly focus on work completed in the last 5 years. We will establish the importance of the individual areas, summarise key reports in the different fields, and offer our insight into how the research community can seek to address and overcome future challenges. For multiple foodborne biosensor examples described in this manuscript, Table 1 provides an overview of many examples named herein, comparing their electrode material, electrode modification, target analyte, detection method, limit of detection (LOD), linear range, and sample composition.

### 2.1. Pathogen Detection

Pathogens are infectious agents capable of causing illness and include fungi, protozoans, bacteria, viruses, and prions [81]. Pathogens include a plethora of microorganisms and molecular-scale infectious agents whose virulence, transmission mode, reproduction mode, etc. vary widely [82,83]. It is because of these complex differences that pathogen sample matrices are intricate in nature, including aerosols, body fluids, and surfaces, and this provides challenges in the optimisation of sample preparation for electrochemical biosensors [84]. While these pathogenic bacteria are too small to be observed by the naked eye, upon culture growth on an agar plate, they can form visible patches/grown patterns. This remains the “gold standard” for bacteria detection, although this diagnostic scheme takes a minimum of 24 h [85,86]. There are in excess of 1400 human pathogens; however, the majority of healthcare-associated diseases are caused by a limited amount [87]. In this pathogenic electroanalytical section, we describe some recent reports towards the electroanalytical detection of some of the most common foodborne pathogens such as *Escherichia coli, Clostridium perfringens, Vibrio cholerae, Staphylococcus aureus, Listeria monocytogenes*, etc.

**Figure 2:**
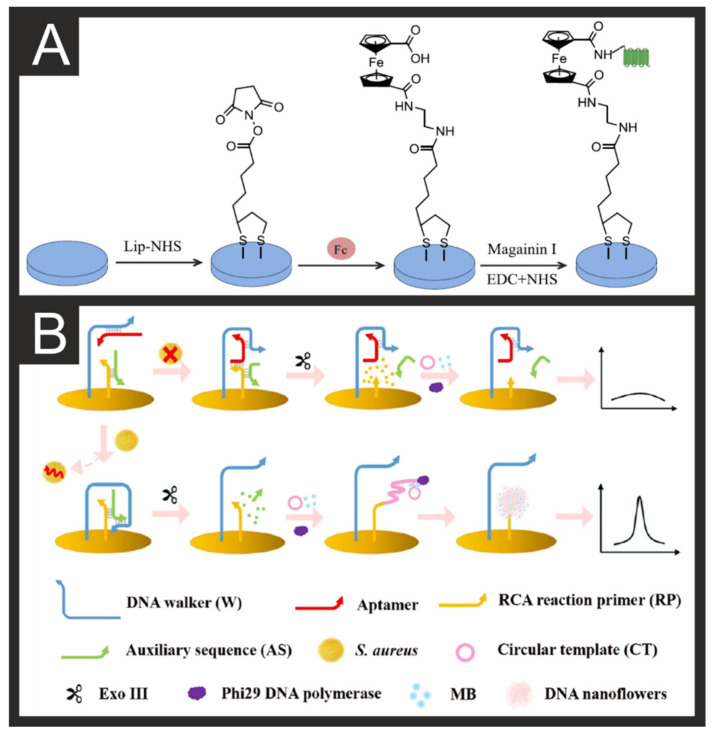
**(A**) Schematic diagram for the construction of a ferrocene-peptide-modified biosensor using a gold macro-electrode. Reproduced with permission from [36]. Copyright Elsevier 2014. (**B**) Schematic representation of the DNA Walker and DNA Nanoflower biosensor for S. aureus. Reproduced with permission from [42]. Copyright American Chemical Society 2021.

*Escherichia coli* is the most common microorganism that affects warm-blooded intestinal organs, and its presence is mainly associated with faecal contamination [29,30,88]. *E. coli* causes diarrhoea, urinary infections, and peritonitis, predominantly in vulnerable people, and it is often used as a microbiological marker for water quality [31]. *E. coli* biosensors are one of the most commonly found in the literature, with various methods and electrodes used such as rotating disk electrodes [32], indium-doped tin oxide [33], glassy carbon electrodes (GCE) [34], and screen-printed electrodes (SPEs) [35]. For example, Kraazt et al. [36] reported a label-free ferrocene-antimicrobial peptide (magainin I) modified gold electrode biosensor, showing its preferential selectivity towards pathogenic strains (O157:H7) with a LOD of 10^3^ cfu/mL. This biosensor solution is based on rapid electrochemical impedance spectroscopy (EIS) biosensor that uses antibacterial peptides as recognition elements for a wide range of Gram+/−pathogens [36]. This biosensor, as shown within Figure 2A, was produced through the deposition of an N-hydroxysuccinimide-based self-assembled monolayer (SAM), followed by coupling to the ferrocene based probe, coupling to the antimicrobial peptide probe, and finally bare surface blocking.

*Clostridium perfringens* is a rod-shaped, Gram-positive, and spore-forming bacteria widely found in meat, dairy, and water that is capable of causing diarrhoea and enteritis necroticans [89,90]. Qian et al. reported the use of ceria (CeO_2_) nanorods as a sensing material towards *C.perfringens* due to its strong adsorption ability towards DNA and low toxicity, compared to CeO_2_ nanoparticles used as control. They immobilised CeO_2_ nanorods onto chitosan (CeO_2_-CHIT), which was then modified upon a glassy carbon electrode (GCE) for the electrochemical detection of *C.perfringens*, which was shown to be possible in pure milk and milk powder samples, achieving a LOD of 7.06 and 1.95 × 10^−15^ mol/L when EIS and differential pulse voltammetry (DPV), respectively. Their label-free biosensors exhibited an easy-to-operate process, highly sensitive, and affordable, with RSD lower than 5% demonstrated within dairy products [37]. Rochelet et al. [38] reported the first amperometric detection of β-D glucuronidase using disposable carbon electrodes as a rapid wastewater analysis method. The authors reported the indirect amperometric quantification of β-D glucuronidase activity and correlated it to the presence of *E. coli.* β-D glucuronidase acts as the electrochemical substrate for GLUase measurement, and the *p*-aminophenol (PAP) released during the enzymatic hydrolysis was monitored by cyclic voltammetry with disposable carbon SPEs achieving a LOD of 10 ng/mL. The amperometric assay was applied to faecal wastewater contamination in raw and treated waters which was applied to turbid sample quantification providing a reliable and decentralised method [38].

A commonly used alternative bio-recognition element to the use of enzymes is antibodies, specific for a certain protein present on the surface of bacteria [91]. Wang et al. [39] reported an antibody-based platform for the pneumococcal surface protein A on *Streptococcus pneumonia*. This bacteria accounted for 95% of the pneumonia cases reported in the pre-antibiotic era and remains a pathogen of concern [92]. The sensor platform utilised DNA tetrahedron nanostructures conjugated onto gold electrode surfaces as their base, with the antibody conjugated on top of that. Through square wave voltammetry, they were able to obtain a LOD of 0.218 ng/mL and applied it to detection in human samples.

*Vibrio cholerae* is the pathogenic agent of cholera, which is a type of life-threatening diarrhoea that can lead to extreme dehydration and death if not rapidly treated, being one of the most rapidly fatal illnesses and therefore of worldwide concern [93,94,95,96]. The use of horseradish peroxidase (HRP) enzyme-based carbon SPE genosensor towards *V. cholerae* was reported by Low et al. [40]. Their strategy uses a double hybridisation strategy (sandwich type) to target *V. cholerae*’s ssDNA to enhance the selectivity and specificity of the detection, without the need for previous heat denaturation or purification, achieving a 100% specificity and LOD of 0.85 ng/μL of *V. cholerae* genomic DNA when coupled to asymmetric PCR amplification.

*Staphylococcus aureus* is one of the most infectious agents in foodborne illness [97,98], with a virulence that includes infective endocarditis, toxic shock syndrome, or osteomyelitis [99]. Wu et al. recently reported the first study of an immunosensor with dual detection and elimination of *S. aureus* in drinks, along with good selectivity, reproducibility, and stability [41]. Their approach is a mussel-inspired scaffold of ε-poly-L-lysine-3,4-dihydroxy benzaldehyde (EPD) that binds to polydopamine (PDA) on a pre-grafted gold macro-electrode. Here, EPD acts as a biomimetic polymer to enhance the immunosensor’s performance with robust binding of the antibody on the electrode’s surface, with pH-responsive properties that allow on-demand ε-poly-l-lysine (ε-PL) delivery to eliminate *S. aureus*. Recently, Cai et al. [42] have reported a DPV-based biosensor for the detection of *S. aureus* with a working range of 60–6 × 10^7^ CFU/mL and LOD of 9 CFU/mL. This platform uses DNA walkers, DNA nanoflowers, and aptamer-based recognition, as shown in Figure 2B, to recognise the presence of the bacteria. DNA walkers can perform repeated movements along a DNA orbit composed of part or all nucleic acids to produce signal amplification [100]. DNA nanoflowers can assemble from localised high concentrations of DNA and do not need full complementary base pairing and have excellent stability [101]. The combination of these two nano-DNA-based systems allows for enhanced signal amplification when the aptamer present is bound to the target bacteria and therefore not interfering with the DNA walker. Using this system, they managed to accurately detect levels of *S. aureus* in spiked lake water, tap water, and honey solutions.

Listeriosis is a bacterial infection caused by *Listeria monocytogenes* that can cause severe illness including sepsis, meningitis, or encephalitis [102]. Listeriosis is mainly a problem from unpasteurised milk (or its derivatives such as cheese and ice cream), vegetables, meats, and fish. Ruan et al. reported the automatic detection of *L. monocytogenes* in milk samples by monitoring the relationship between oxygen consumption and the concentration of the analyte by cyclic voltammetry, where the oxygen reduction decreases/disappears when *L. monocytogenes* proliferates when using a gold disk macro-electrode (GDE) in a Listeria enrichment broth (LEB) [103].

The presence of bacteria inside food and drink products is of vital concern, but these microorganisms can also release toxins into these substances, which is where our focus now turns.

### 2.2. Toxins/Mycotoxins

Toxins are harmful compounds produced by living cells or organisms such as bacteria, fungi, or algae, whose composition varies from small molecules to large biomolecules such as peptides or proteins [104,105]. Toxins are usually divided between exo- and endotoxins depending on whether they are excreted by an organism or are a structural part of the cell. In nature, toxins can have two primary functions—predation and defence—to kill a potential meal or to discourage the action of a third party (predator). Another method of classification can be in accordance with the location of the body that they affect the most, such as hemotoxin (blood), phototoxin (light related), necrotoxin (damages tissue), or neurotoxin (nervous system). For example, the botulinum toxin is a spore (endotoxin) produced by *Clostridium botulinum* that can block neurotransmitter metabolic pathways that cause muscle and lung paralysis, double vision, and muscle weakness in humans, named botulism [106,107]. Botulinum toxin can be produced from foods with pH > 4.5 and sufficient moisture, such as homemade tinned and fermented foods with long shelf life. Another example is Staphylococcal enterotoxins (SEs) secreted by *Staphylococcus aureus* (*S. aureus*), *S. hyicus*, and *S. intermedius* that are a major cause of food poisoning, with nausea, violent vomiting, and diarrhoea as main symptoms and typically occur after ingestion of dairy or processed meat that has been improperly handled or stored at elevated temperatures [108]. Many other common bacterial foodborne toxins are cholera toxin (Ctx) from *Vibrio cholerae*, Shiga Toxin from *Shigella dysenteriae*, and *E. coli* O157:H7, and CPE enterotoxin from *Clostridium perfringens* [109].

**Figure 3:**
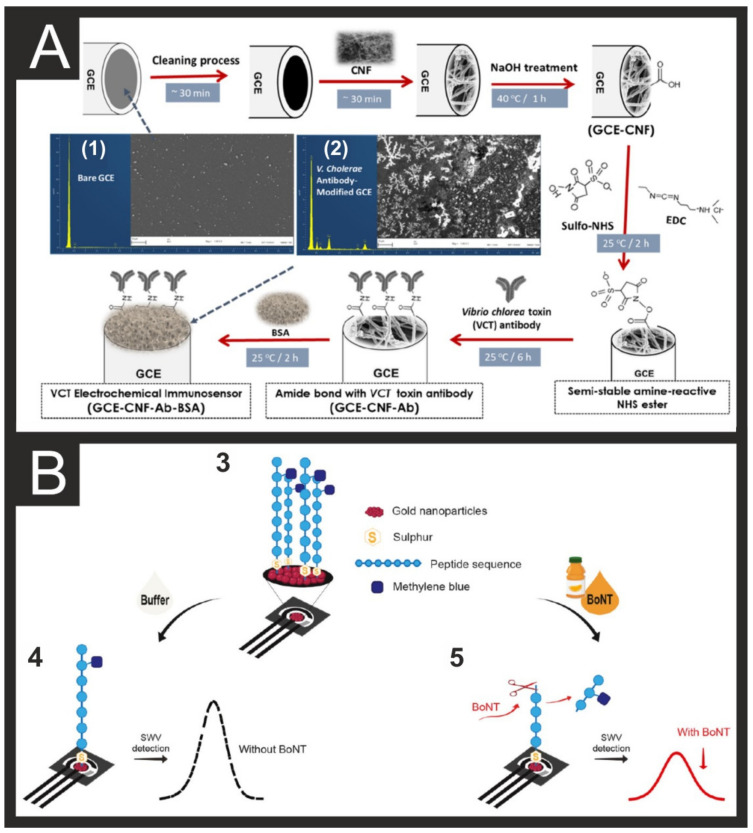
(**A**) Experimental protocol (and time needed) for the fabrication and sensing mechanism of an electrochemical immunosensor for VCT. Inset (1) shows typical SEM and EDX of a bare GCE, while (2) shows SEM and EDX of *V. cholerae* antibody-modified GCE. Reproduced with permission from [43]. Copyright American Chemical Society 2020. (**B**) Experimental protocol for the BoNT detection using the signal-off method, including a representation of the peptide-modified paper-based sensor (3), signal obtained before analyte presence (4), and signal obtained after analyte presence (5). Reproduced with permission from [45]. Copyright Elsevier 2021. Algae/Algal toxins.

A recent example of an electrochemical immunosensor for Ctx in water samples from Ozoemena et al. utilises electrospun carbon nanofibers (CNFs) on a GCE, with the α-Ctx antibody covalently immobilised using carbodiimide chemistry for amide bond formation (Figure 3A) [43]. Their electrochemical response is based on the suppression of the electrical current, followed by EIS or square wave voltammetry (SWV), where the use of the ferri-/ferro-redox probe enhances the sensitivity of the immunosensor to detect Ctx. This immunosensor exhibited excellent sensitivity, selectivity, and regenerability; LOD of ca. 1.2 × 10^−13^ g/mL and limit of quantification (LOQ) of ca. 1.3 × 10^−13^ g/mL [43]. Another example of a novel electrochemical sensor for the detection of botulinum neurotoxin (BoNT/E) in milk and orange juice was reported by relying on graphene nanosheet-s-aryldiazonium modified GC as a sensing platform, and enzyme-induced silver nanoparticles (AgNPs) deposited on gold nanoparticles (AuNPs) as a signal amplifier. The authors demonstrated a highly sensitive and specific electrochemical immunosensor for the detection of BoNT/E based on an enzyme-AuNPs accelerated silver deposition with ordered graphene nanosheets to amplify the signal, increase the surface area and provide beneficial antifouling properties [44]. Another system from Caratelli et al. [45] for the detection of both BoNT/A and BoNT/C with LODs of 10 pM. A peptide labelled with methylene blue is immobilised onto a paper-based electrode surface modified with AuNPs, as depicted in Figure 3B. The BoNTs, when present, cleave the synthetic peptide, removing the methylene blue and causing significant decreases in the measured square-wave signal. Last, a zearalenone (ZEA; mycotoxin in cereals) sensor was developed using carbon nanotubes (SWCNTs) on a screen-printed electrode (SWCNT-SPE) for the voltammetric (differential pulse adsorptive stripping; DPASV) determination, with a LOD of 5.0 × 10^−9^ M in cornflake samples. The use of SWCNTs here enhances the sensitivity attained by DPV, lowers the LOD over a wide range of concentration, and shows antifouling properties [46]. Another issue for the safety of especially drinks, in addition to food that originates from water-based environments, is the presence of algae or algal toxins, which we will discuss next.

**Figure 4:**
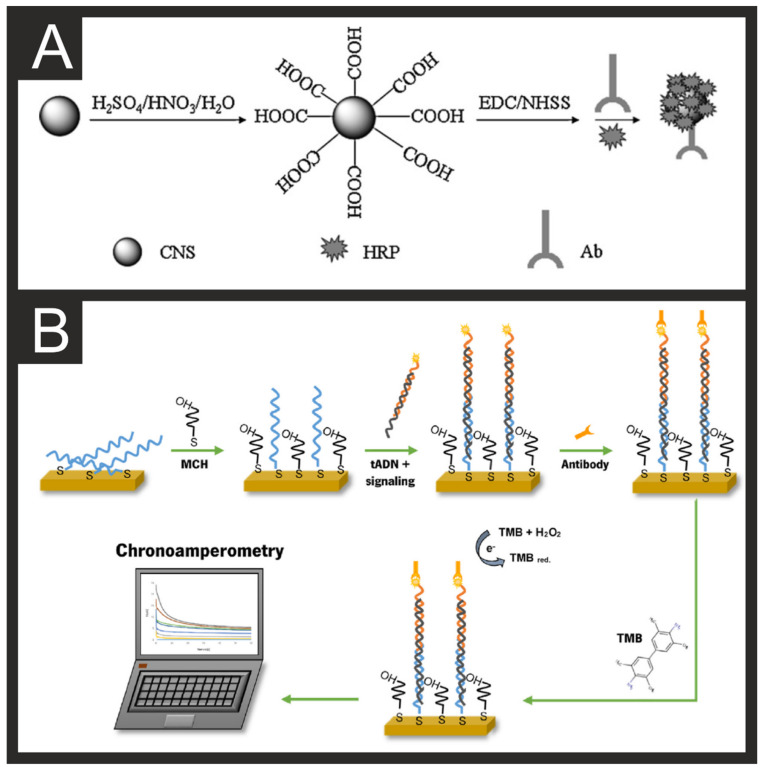
(**A**) Schematic illustration of the carboxylation procedures of CNS and the subsequent HRP-CNSs-Ab conjugation. Reproduced with permission from [47]. Copyright Elsevier 2013. (**B**) Schematic for the development of the electrochemical genosensor for *A. minutum*. Reproduced with permission from [49]. Copyright Elsevier 2021.

Different species of cyanobacteria or dinoflagellates are capable of producing a number of toxins and often cause episodes of harmful algal blooms (HABs) in fresh or marine water bodies from eutrophication occurrence arising from human activities [110,111,112,113]. These anthropogenic activities usually include agricultural and urban waste, industrial manufacture, and global warming [114,115]. HABs can create a change in colour, odour, and taste due to the production of algae toxins, some of which have harmful effects on humans. Examples of harmful algae toxins that can occur in drinking and recreational water are microcystins, and cylindrospermopsin (hepatotoxin), anatoxins, and brevetoxins (neurotoxins), and certain lipopolysaccharides and lyngbyatoxin (dermatoxins) [116]. Algae toxins are often secondary metabolites that belong to a variety of class compounds such as cyclic peptides, alkaloids, and lipopolysaccharides [113]. A recent example of a cyanotoxin drinking water outbreak was the famous case of Lake Erie in Toledo (OH, USA), where tap water was not suitable for human consumption during the summer of 2013–2014 due to the high presence of cyanotoxins in their supply [117,118]. Antibodies, aptamers, carbohydrates, and antimicrobial peptides have been used as biorecognition elements for electrochemical biosensors [119]. Due to the small size of algae toxins (MW < 1 KDa), competitive immunoassays are often employed for sensing purposes, in which the working electrode surface would be linked to algal toxins and incubated with antibodies, followed by the addition of the algal unknown samples. The unknown algal toxins would then compete or displace the ones immobilised at the electrode’s surface, offering enhanced electrochemical performance, compared to those based on antibodies as biorecognition elements. An example of these types of competitive solutions is the one reported by Quan et al. towards the detection of Microcystin-LR (MC-LR) in water samples [47]. They reported a graphene sheet-chitosan (GS-CS) nanocomposite on a glassy carbon electrode that acts as a scaffold for MC-LR detection. As signal reporters, horseradish peroxidase (HRP), carbon nanospheres (CNS), and antibody conjugates were used, Figure 4A. Their clever design increased the active area and sensitivity by using CNS, which offers a 3D structure and good biocompatibility while offering enhanced electron transfer properties that achieved a LOD of 0.016 μg/L and variation coefficients around 1% for their proposed immunosensor [47].

Aptamers are single-stranded oligonucleotides capable of binding to a specific target molecule that belong to a group of single-stranded (ss) DNA/RNA molecules [120]. Aptamers are of small size and offer higher affinity, stability, and specificity to target molecules than some antibodies and can be tailored to contain certain terminal moieties to bind to the surface of the electrode (thiol, amino, disulphide, etc.) [121,122,123], although their stability and shelf life is still a challenge for their large-scale commercialisation and use. It has also been reported that the use of Mg^2+^ and pH changes can enhance the binding affinity to algal toxins by controlling the secondary conformation of the aptamer and increase their stability [124,125]. A recent example of detecting important biotoxins domoic acid (DA) and okadaic acid (OA) in mussels was reported by Nelis et al. [48]. They functionalised carbon-black-modified carbon SPEs (drop-casted CB-SPE) with protein conjugates specific for DA and OA and utilised them in indirect competitive DPV immunosensors. Through this approach, the authors achieved LODs of 1.9 ng/mL and 0.18 ng/mL in mussel extract samples.

The dinoflagellates from the *Alexandium* genus can include some of the most toxic species [126]. Morais et al. [49] have reported a disposable electrochemical genosensor for the detection of *Alexandrium minutum* through the immobilisation of a DNA-capture probe onto a screen-printed gold electrode (SPGE) targeting a specific coding sequence (Figure 4B). The chronoamperometric detection achieved a linear range of 0.12–1.0 nM and a LOD of 24.78 pM, with RSD < 5.2 % shown to be possible.

### 2.3. Allergen Examples

**Figure 5:**
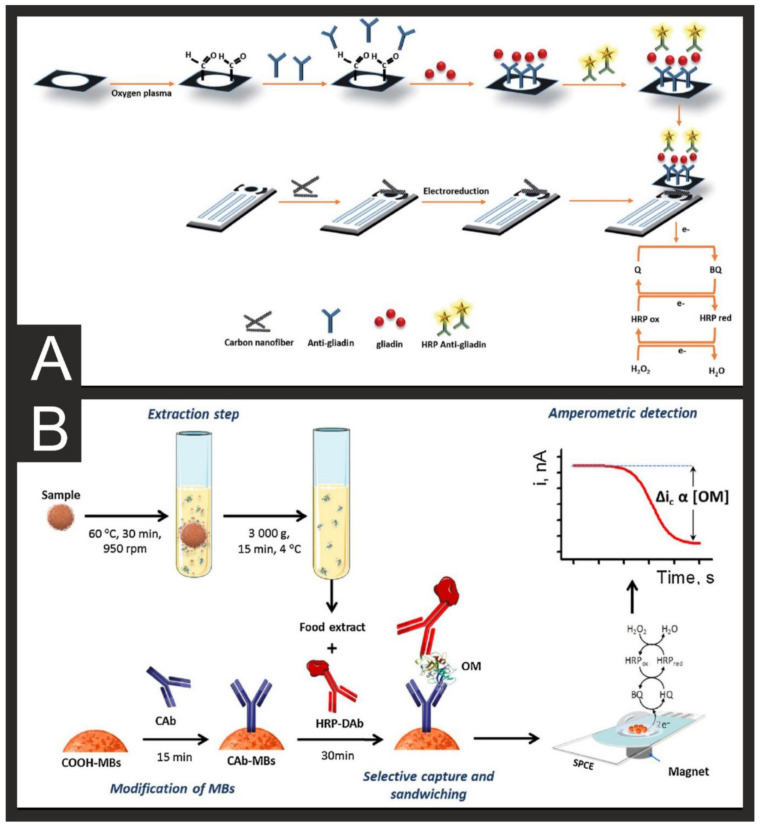
(**A**) Schematic representation of the electrode modification and gliadin determination procedures. Reproduced with permission from [50]. Copyright Royal Society of Chemistry 2019. (**B**) Schematic display of the fundamentals involved in the immunosensing platform developed for OM determination. Reproduced with permission from [51]. Copyright Elsevier 2018.

Allergens are a type of antigen that trigger an abnormal immune response against an otherwise harmless substance in the body; these types of reactions are commonly known as allergies and are believed to affect around 3% of adults and 10% of children in industrialised countries [127]. These allergies can be divided into toxic or nontoxic, depending on the individual sensitivity, and can be further subdivided into immunological (food allergies) or non-immunological (food intolerances) [128,129]. Overall, 14 ingredients have been included in the European Union list of allergenic food ingredients—eggs, milk, peanuts, nuts, gluten-containing cereals, lupin, soybeans, celery, mustard, sesame, fish, crustaceans, molluscs, and sulphites [130,131,132]. Although detailed food labelling is required in most countries, cross-contamination, adulteration, or fraud of undeclared allergens often occurs. Pereira et al. [50] reported a disposable and low-cost biosensor strategy towards gliadin in common flour samples. Gliadin is a protein component of gluten which provides the rising ability during baking and is present in wheat and many other cereals from the grass genus Triticum. Their elegant solution uses a carbon SPE modified with carbon nanofibers, which are coupled to a paper immunoaffinity platform for the gliadin sensing in flour samples (Figure 5A). The choice of carbon nanofibers herein allows an increase in electron transfer efficiency and in the electroactive area, enabling the determination of low levels of analyte. This paper platform uses a covalently functionalised micro-zone, with specific anti-gliadin antibodies placed for the voltammetric detection of gliadin, reporting a LOD of 0.005 mg/kg and a 4.11% variation coefficient for a 20 μg/kg gliadin sample. Their activated cellulose paper offers hydroxyl groups for bioconjugation purposes, which allows the covalent bonding of the amino groups from the antibodies. The use of an HRP saturation measurement to quantify the available sites not previously occupied by antibodies will catalyse the catechol–benzoquinone reaction in the presence of H_2_O_2_, generating a current response that is inversely proportional to the amount of immobilised antibody [50]. Another recent example of an allergen electrochemical biosensor is the one reported by Pingarron et al. [51] for ovomucoid allergen (OM; egg white allergen) by using magnetic bioconjugates captured on a carbon SPE surface to perform an amperometric detection in the presence of hydroquinone and H_2_O_2_. Their immunoplatform involves the selective capture of sandwich antibody-target analyte-HRP labelled detector antibody sandwich onto a carboxylic acid-functionalised magnetic bead (HOOC-MBs), as shown in Figure 5B, exhibiting a LOD of 0.1 nm/mL for the determination of OM in unprocessed eggs and flour and baked bread [51]. Another example targeting egg allergen (ovalbumin, OVA), this time in wine samples, has been reported by Baldo et al. [52]. In the wine industry, egg white can be utilised as a fining agent to assist in the removal of tannins. The authors report a very similar sandwich-based immunoassay to that described above using OVA-specific antibodies and HRP decorated onto magnetic beads (Figure 5). An array of 8 SPEs modified with poly(diallyldimethylammonium chloride) and graphene oxide, which aided with the biomodification, was modified with specific antibodies through carbodiimide coupling. The modified MBs were mixed with the sample and then incubated onto the electrode. If the target was present, they would bind to the surface Abs and provide an enhanced electrochemical signal through the HRP.

### 2.4. Veterinary Drugs

Antimicrobial, antiparasitic, and growth promoters are normally included in the veterinary drugs category due to their work in treating and promoting animal growth and feed efficiency and preventing diseases [133]. Their quantification is of public concern due to the possible presence of these drugs in animal-derived foods, which might cause harmful effects to the final food product if their quantities are higher than the maximum residue limits (MRL) defined on the basis of food safety [134]. Common antibiotics used in veterinary practice often include sulphonamides, lincosamides, nitrofurans, etc. [135,136,137]. From these compounds, trace residues present in the final food (milk, meat, egg, honey, etc.) product can cause serious harm to humans [138]. An added risk recently discovered is that the presence of these antibiotics in food can also trigger antimicrobial resistance later in the food chain if inefficient antibiotic therapies emerge [139,140]. There are many examples of various electrochemical sensing platforms for veterinary drugs found throughout the literature, such as oxyclozanide [53], monensin [54], and tetracycline [55].

**Figure 6:**
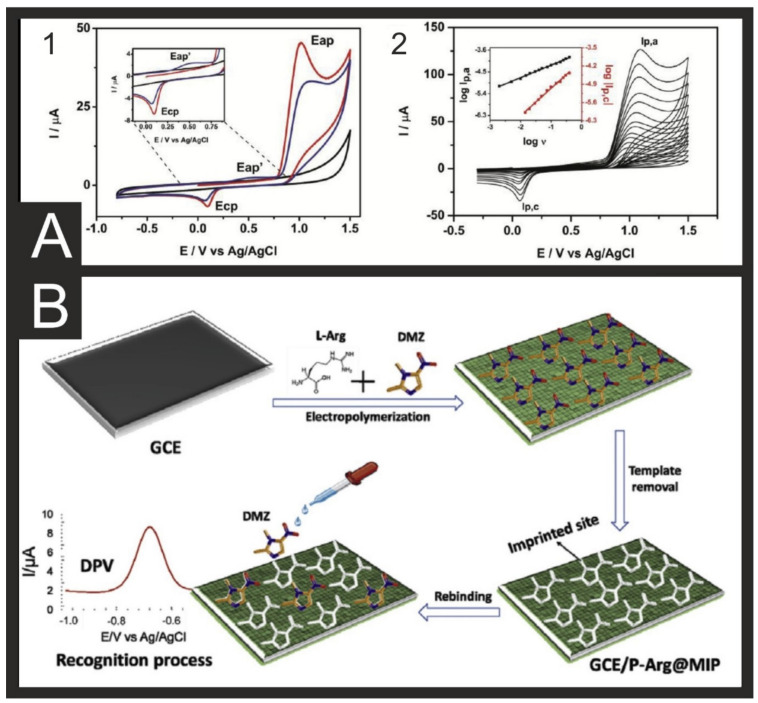
(**A**) (1) Cyclic voltammograms (scan rate: 50 mVs^−1^) recorded using a GCE in a BR buffer (0.1 M, pH = 7.0) in the absence (black line) and presence of 0.4 mM xylazine (red line—first scan, blue line—second scan). Shown in the insert is the zoom plot of the anodic process on the second scan. (2) Scan rate study (2–400 mVs^−1^) of the above solution. Inserted is the plot of the log of the peak current versus the log of the scan rate. Reproduced with permission from [56]. Copyright Elsevier 2019. (**B**) Schematic for the formation of the PAG MIP sensor for the detection of DMZ. Reproduced with permission from [60]. Copyright Elsevier 2020.

As an example of a non-approved substance for human use in food, xylazine is a clonidine derivative that is an analgesic and sedative for animals [140,141]. In humans, xylazine causes diarrhoea and drowsiness, acting on the central nervous system, and if long exposure occurs, it can develop a drug dependence that might lead to depression, sleepiness, and lowered respiratory rates [142]. Xylazine can be detected electrochemically through its irreversible oxidation peak at approximately +0.9 V (Figure 6A) [56]. Saisahas et al. recently reported a simple portable electrochemical sensor for xylazine based on drop-casted graphene nanoplatelets (GNPs) on carbon SPEs for the voltammetric determination in spiked alcoholic and non-alcoholic drinks. Their choice of GBPs allowed enhanced adsorption and a unique electrochemical profile, which is based on adsorptive stripping voltammetry (AdSV) [57]. Zhang et al. reported an ingenious use of commercial personal glucose meters (PGM) coupled with a novel sensitive method for ampicillin detection in milk. This method uses magnetic beads (MBs), combining an ampicillin aptamer as a recognition element and streptavidin as a linking agent, for the indirect relationship between ampicillin and the sucrose to glucose hydrolysis, achieving a LOD of 2.5 × 10^−10^ mol/L in milk [58].

Dimetridazole (DMZ) is used to treat protozoal and bacterial infections and is commonly added to poultry feed. Residues of this, however, can produce carcinogenicity, genotoxicity, and mutagenicity in humans [143]. Recently, Umesh et al. [59] reported a biosensing platform for the detection of DMZ in milk, pigeon meat, and eggs based on Se nanorods capped with Co_3_O_4_ nanoflowers decorated onto graphene oxide. This system combined these materials together to produce enhanced electron transfer and a higher conductivity and are deposited onto the surface of the GCD through simple drop-casting. They achieved a wide linear range of 0.02–83.72 µM, a LOD of 3.4 Nm, and excellent recoveries in real samples. Another example for the detection of DMZ was reported by Ali et al. [60], who used a poly-arginine (PAG) based MIP on a GCE (Figure 6B). The PAG MIP was formed directly onto the surface of the GCE through electrodeposition in the presence of DMZ using cyclic voltammetry. Upon rebinding of the DMZ with the MIP, increases in the DPV signal were obtained leading to a linear range of 0.1 nM–100 µM and a LOD of 0.1 nM.

### 2.5. Pesticides

Herbicides, insecticides, and fungicides are commonly used within food production to control their pests and increase production [144]. These pesticides, although toxic for their targets, can also be harmful to other animals and humans, causing carcinogenicity, infertility, neurological diseases, respiratory problems, etc. [145]. As an example, an organophosphorus and carbamate pesticides biosensor was recently reported for olive oil samples, comparing the different protocols for AChE deposition on CB drop-casted on carbon SPEs, using laser-induced forward transfer (LIFT) technique to modify the electrodes [61]. The use of LIFT minimises the amount of deposited material, reduces the cost, and produces high spatial resolution allowing the biofunctionalisation of small-area surfaces [146,147]. Their olive oil pesticide sensors exhibited LODs of 0.6 and 0.4 × 10^−9^ mol/L for carbofuran and chlorpyrifos, respectively [61]. Della Pelle et al. also reported a CB-SPE voltammetric sensor for carbofuran, isoprocarb, carbaryl, and fenobucarb in grain samples with comparable results to that of UPLC-MS/MS [62]. Validation against these standard laboratory-based techniques is vital in providing confidence in the electrochemical platforms for potential end users and legislation creators. Govindasamy et al. also reported the use of graphene oxide nanoribbon (GONRs) modified SPEs for a disposable real-time detection of methyl parathion in broccoli, beetroot, tomato, and Ugli fruits [63]. GONR-SPEs exhibited improved electrocatalytic ability towards methyl parathion in comparison with the CNT counterparts, due to the rich edge chemistry and abundant functional groups, higher area-normalised edge-plane structures, and chemical active sites [63].

**Figure 7:**
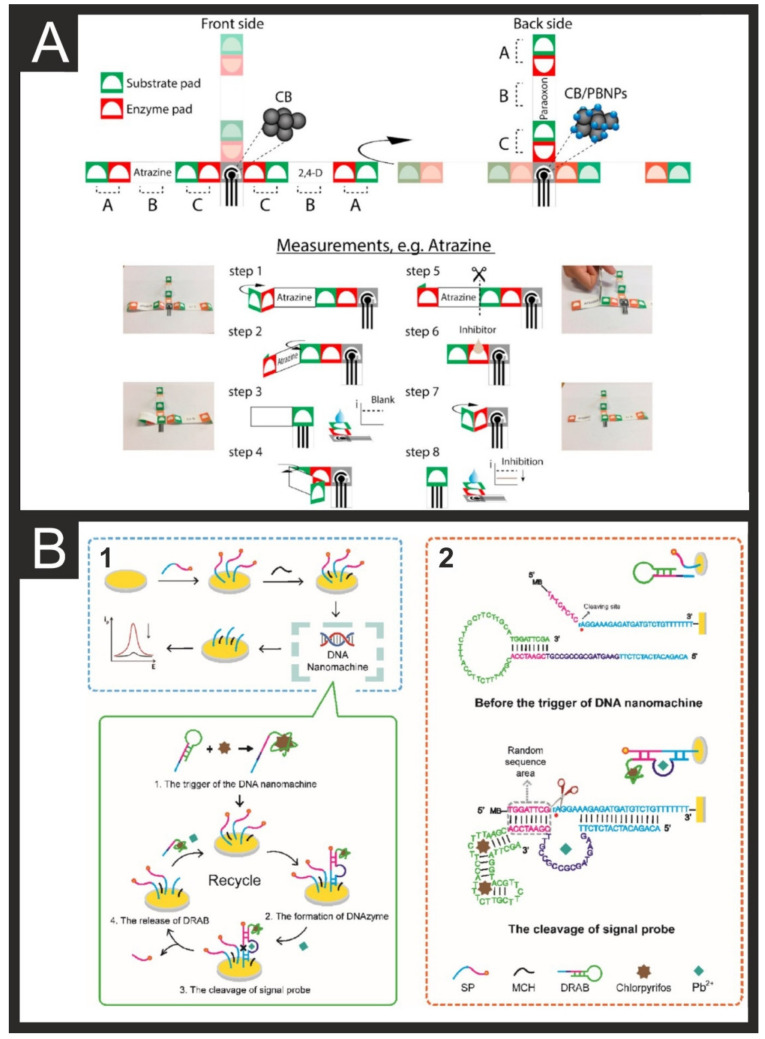
(**A**) Schematic representation and photographs of the configuration of the paper-based platform and measurement procedure. (**B**) The principle of DRAB-based electrochemical biosensors (1) Modification and detection procedure of the proposed sensor. (2) The secondary structure of the DRAB.

An interesting approach to the detection of pesticides is the origami-paper-based sensors presented by Arduini et al. [64]. In this work, they utilised office-paper-based carbon SPEs alongside multiple filter paper pads for the loading of enzymes and enzymatic substrates. A hydrophobic wax before printing, creating hydrophilic zones, defines the electrode areas. Thereafter, the working electrode was modified with a carbon black/Prussian blue nanoparticle dispersion. The pads on either side of the SPE are loaded with the appropriate enzymes and substrates. When samples testing is required, the appropriate outer pads are folded onto the SPE surface before the droplet is added and analysis can occur (Figure 7A). This was applied to the detection of paraoxon, 2,4-dichlorophenoxyacetic acid, and atrazine, whereby the presence of these analytes inhibits the immobilised enzymes and substrates. This was monitored using chronoamperometry and used to evaluate the analytes at the ppb level. Similar paper-based devices have been recently applied to the detection of organophosphorus pesticides in soil with a LOD of 1.3 ng/mL, with this system validated against LC-MS lab-based techniques [65]. Organophosphorus pesticides are some of the most commonly used throughout the agriculture industry to control pests and diseases and increase crop production. As such, there have been various electrochemical biosensors designed for their detection utilising different recognition elements such as antibodies [66], nanozymes [67], enzymes [68], and aptamers [69]. The aptamer-based system reported by Fu et al. utilised reduced graphene oxide (rGO) as a base for the immobilisation of copper nanoparticles (CuNPs) and the aptamer element. The use of micro and nanomaterials in biosensor development has been regularly used to increase the surface area, conductivity, and analytical performance [148], such as graphene oxide [149], reduced graphene oxide [150], and carbon nanotubes [151]. A recent example utilising a mix of materials was reported by Renganathan et al. [70], where they used palladium nanoparticles adorned with boron nitride for the electrochemical detection of paraoxon ethyl (PXL). Boron nitride provides a high specific surface area and an effective pathway for mass transport, with the nanoparticles improving the electrochemical performance. This allowed for the direct electrochemical detection of PXL, through linear sweep voltammetry, with a linear range of 0.01–210 µM and a LOD of 3 nM. Finally, a novel methodology for dual detection of the pesticide chlorpyrifos and heavy metal ions. Wang et al. [71] utilised a dual-recognition aptazyme beacon (DRAB), which contains both the aptamer specific for chlorpyrifos and an enzyme strand of Pb^2+^ DNAzyme. The aptamer is bound to the 3′ terminus of the enzyme strand preventing it from forming the activeated DNAzyme. In the presence of the analyte chlorpyrifos, the DNA nanomachine is activated and DRAB unfolded (Figure 7B). Upon the binding of Pb^2+^, the DRAB is catalysed and the signal probe is cleaved from the surface, resulting in a decrease in the DPV signal. This was applied to the detection of the analytes in fresh fruit and vegetable samples that had been turned into a puree. This is a good example of analysis in complex real samples, which remains a challenge in this field. As seen throughout this section, the majority of pesticide detection has been performed on water samples, typically looking for contamination in open water sources.

## 3. Future Trends

Currently, and due to the 2019 SARS-CoV/COVID pandemic, the general public has seen the importance of rapid, reliable, and robust diagnostic kits for the early detection of diseases by using rapid serological tests. However, the need for rapid on-site monitoring does not stop at the management of pandemics, but it is applicable to healthcare and forensic biomarkers, environmental monitoring, and food safety. The benefit of obtaining fast results in decentralised environments makes these tests part of an on-growing trend for their rapid turnarounds and ability to mitigate consequences and harmful exposure to humans and the environment.

The fundamental concept of point-of-care (PoC) and/or on-site testing devices is to carry out the test in the most appropriate, comfortable, and immediate way without extensive sample preparation, expensive kits, or specialised experimentalists. Based on this, electrochemical biosensors offer most of the ideal requirements for their miniaturisation for PoC applications. One of the main challenges that experimentalists encounter when developing a biosensor for food safety is the application to real samples. One can easily find reports in the literature showcasing contaminants, pathogens, and allergens biosensors; however, their application to real-world samples is often limited. As summarised in Table 1, most of the real-world applications of these food safety biosensors are in water or drink samples. Therefore, there is a need for improving the performance of these devices, particularly in complex matrices, in order to transition from laboratory-based methods to PoC solutions.

Lastly, with the recent increase in computing power and the ever-decreasing cost of computers, researchers are moving towards a trend of design and development of experiments (DoW), artificial intelligence (AI), cloud-based and Internet-of-Things (IoT) solutions that, when coupled with extensive databases and complex algorithms and chemometric studies, will boost the sensing capabilities of PoC devices which eventually will also lead to new scientific discoveries in terms of epidemiology, environmental monitoring and remediation, water sanitation, early diagnostics, etc. In summary, we encourage electrochemists to explore and invest in developing a new generation of intelligent databases and algorithms capable of resolve matrix- and real-sample problems by applying computer modelling and AI.

## 4. Conclusions

Food and drink safety is of critical importance to the health and well-being of the human population. The rapid, in situ detection of possible contamination is becoming increasingly important as the importing and consumption of products from all areas of the globe continue to grow. Electrochemical sensing platforms offer an alternative route to solving this challenge due to their rapid testing, portability, and low cost. This has been demonstrated through the plethora of literature articles published on the detection of a wide variety of possible targets. There are numerous different strategies employed by researchers with examples of direct detection of redox-active targets, use of enzyme catalysis, antibody sandwich assays, etc. Although these sensing platforms have shown excellent performance and promise towards their application, more research must be conducted to overcome future challenges such as sample preparation and interferent substances for detection in the more complex matrices. We also recommend that authors consistently validate their results against the commonly used laboratory standards to raise confidence in the results. This will help to gain trust in the results from food and drink producers, policymakers, and consumers, leading to enhanced chances of commercialisation.

## Figures and Tables

**Table 1:** An overview comparing the different electrodes and their modifications for detection of different analytes relevant to food safety alongside the limits of detection and linear ranges achieved and the composition of samples that were tested.

Electrode Materials	Electrode Modification	Target Analyte	Detection Method	Limit of Detection	Linear Range	Sample Composition	Reference
ITO/GCE	Cu@Au labelled Abs/Nafion and Hg	*E. coli*	ASV	30 CFU/mL	50–50,000 CFU/mL	Surface water	[29]
SPE	Abs	*E. coli*	CA	10^3^ CFU/mL	10^3^–10^7^ CFU/mL	Water	[30]
Graphite	Teflon/tyrosinase	*E. coli*	CA	10 CFU/mL	10–10^7^ CFU/mL	Drinking water	[31]
Ni disk	NiOOH/Ni(OH)_2_	*E. coli*	CA	10^4^ CFU/mL	6.4 × 10^4^–3.3 × 10^9^ CFU/mL	Water	[32]
ITO	CNT	*E. coli*	CC	2 × 10^3^ CFU/mL	10^5^–10^7^ CFU/mL	Drinking/Tap water	[33]
GCE	rGO-PVA/AuNP/Aptamer	*E. coli*	DPV	9.34 CFU/mL	9.2–9.2 × 10^8^ CFU/mL	Tap water, milk, meat	[34]
SPE	AuNP/Abs	*E. coli*	EIS	15 CFU/mL	10^1^–10^6^ CFU/mL	Water	[35]
Au	SAM/FcD/Peptide	*E. coli*	EIS	10^3^ CFU/mL	10^3–^10^7^ CFU/mL	Water	[36]
GCE	dsDNA/CeO_2_/ CHIT	*C. perfringens*	EIS	1.95 fM	10 fM–100 nM	Dairy products	[37]
SPE	N/A	*E. coli*	CV	10 ng/mL	10–1000 ng/mL	Wastewater	[38]
Au	DNA-TH/Abs	*S. pneumoniae*	SWV	0.093 CFU/mL	5–100 CFU/mL	Nasal, mouth and axilla samples	[39]
SPE	ExtrAvidin^®^/ VHMR	*V. cholerae*	CA	0.95 ng/µL	0.49–15.6 nM	Water	[40]
GCE	PDA/EPD/Abs	*S. aureus*	DPV	28.55 CFU/mL	10^4^–10^10^ CFU/mL	Milk	[41]
Au	DNA walker/RP	*S. aureus*	DPV	9 CFU/mL	60–6 × 10^7^ CFU/mL	Water, honey	[42]
GCE	CNF/Abs	*V. cholerae*	EIS	1.2 × 10^−13^ g/mL	10^−13^–10^−5^ g/mL	Water samples	[43]
GCE	Ph-PhNH_2_/GNS/Abs	BoNT/E	LSV	5 pg/mL	0.01–10 ng/mL	Orange juice, milk	[44]
SPE	AuNPs/Peptide	BoNT/A&C	SWV	10 pM	0.01–1 nM	Orange juice	[45]
SPE	SWCNT	ZEA	DPASV	5 nM	0.0025–1 µM	Cornflakes	[46]
GCE	GS/CHIT	Microcystin-LR	DPV	0.016 µg/L	0.05–15 µg/L	Water	[47]
SPE	CB/ovalbumin	DA/OA	DPV	1.9/0.18 ng/mL	4–34/0.35–3.9 ng/mL	Mussel extract	[48]
Au-SPE	DNA-capture probe	*A. minutum*	CA	25 pM	0.12–1 nM	Ocean sample	[49]
SPE	CNF/Abs	gliadin	CA	0.005 mg/kg	0–80 µg/kg	Flour samples	[50]
SPE	MBs/Abs	ovomucoid	CA	0.1 ng/mL	0.3–25 ng/mL	Eggs, flour, bread	[51]
SPE	GO/MBs/Abs/ HRP	ovalbumin	CA	0.2 fg/mL	0.01–10 pg/mL	Wine	[52]
CPE	-	oxyclozanide	SWASV	17.42 µg/L	0.058–4 mg/L	Pharmaceutical formulation	[53]
GCE	Zn/Ni-ZIF-8 800/G/AuNp/Abs	monensin	DPV	0.25–100 ng/mL	0.11 ng/mL	Milk	[54]
Au	MBs	tetracycline	EIS	1.2 pg/mL	0.1–1000 pg/mL	Honey	[55]
GCE	-	xylazine	DPV	120 nM	0.5–256 µM	Pharmaceutical formulation/urine	[56]
GCE	GNP	xylazine	ASV	0.1 mg/L	0.4–6 mg/L	Beverages	[57]
PGM	MBs/Aptamer	ampicillin	-	0.25 nM	0.25–100 nM	Milk	[58]
GCE	Se-Co_3_O_4_/GO	dimetridazole	DPV	3.4 nM	0.02–83.72 µM	Pigeon meat, eggs	[59]
GCE	P-Arg-MIP	dimetridazole	DPV	0.1 nM	0.1 nM–10 µM	Egg, milk, honey	[60]
SPE	CB/acetylcholinesterase	Carbofuran chlorpyrifos	CA	0.6 nM 0.4 nM	1.1–23 nM 0.7–14 nM	Olive oil	[61]
SPE	CB	Carbofuran Isoprocarb Carbaryl fenobucarb	DPV	0.048 µM 0.049 µM 0.079 µM 0.80 µM	0.1–100 µM	Wheat and maize	[62]
SPE	GONRs	Metyl parathion	CA	0.5 nM	100 nM–100 µM 100–2500 µM	Tomato, beetroot, broccoli	[63]
SPE	CB/PB/Enzyme	Paraoxen 2,4-dichlorophenoxyacetic acid atrazine	CA	2–20 ppb 100–600 ppb 10-100 ppb	2 ppb 50 ppb -	River water	[64]
SPE	CB/PB/BChE	paraoxon	CA	1.3 ng/mL	0.0013–3 µg/mL	Soil, fruit, vegetables	[65]
SPE	AuNP/PB/Abs	OPs	DPV	0.003 ng/mL	1.82 × 10^−3^–3.29 × 10^4^ ng/mL	Cabbage	[66]
ITO	MnNS	OPs	DPV	0.025 ng/mL	0.1–20 ng/mL	Pakchoi	[67]
ITO	MB/ZIF-8/AChE	paraoxon	DPV	1.7 ng/mL	20–4000 ng/mL	Apple, aubergine	[68]
SPE	rGO-CuNPs/Aptamer	Profenofos Phorate Isocarbophos omethoate	DPV	0.003 nM 0.3 nM 0.03 nM 0.3 nM	0.01–100 nM 1–1000 nM 0.1–1000 nM 1–500 nM	Spinach, rapeseed	[69]
GCE	PdNPs/BN	Paraoxon ethyl	LSV	3 nM	0.01–610.5 µM	River water	[70]
Au	DRAB	Chlorpyrifos Pb	DPV	0.178 nM 0.034 nM	0.5–500 nM 0.1–500 nM	Apple, orange, cabbage	[71]

ITO: indium-doped tin oxide; ASV: anodic stripping voltammetry; Abs: antibodies; GCE: glassy carbon electrode; SPE: screen-printed electrode; CA: chronoamperometry; CC: chronocoulometry; CNT: carbon nanotube; rGO: reduced graphene oxide; PVA: poly(vinyl alcohol); AuNP: gold nanoparticles; DPV: differential pulse voltammetry; EIS: electrochemical impedance spectroscopy; SAM: self-assembled monolayer; FcD: ferrocene derivative; dsDNA: double-stranded DNA; CHIT: chitosan; CV: cyclic voltammetry; DNA-TH: DNA tetrahedron; SWV: square-wave voltammetry; VHMR: target PCR amplicon; PDA: polydopamine; EPD: ε-poly-L-lysine-3,4-dihydroxy benzaldehyde; RP: RCA reaction primer; CNF: carbon nanofibers; BoNT/E: botulinum neurotoxin-E; LSV: linear sweep voltammetry; GNS: graphene nanosheets; ZEA: zearalenone; DPASV: differential pulse adsorptive stripping voltammetry; SWCNT: single-walled carbon nanotubes; GS: graphene sheets; DA: domoic acid; OA: okadaic acid; MBs: magnetic beads; HRP: horseradish peroxidase; GO: graphene oxide; CPE: carbon paste electrode; SWASV: square-wave adsorptive stripping voltammetry; G:graphene; Zn/Ni-ZIF-8 800: Zinc/Nickel-zeolitic imidazolate framework-8; GNP: graphene nanoplatelets; PGM: personal glucose meter; MIP: molecularly imprinted polymer; P-Arg: polyargenine; CB: carbon black; GONRs: graphene oxide nanoribbons; PB: Prussian blue; BChE: butyrylcholinesterase; Ops: organophosphorus pesticides; MnNS: manganese dioxide nanosheets; MB: methylene blue; AChE: acetylcholinesterase; BN: boron nitride; DRAB: dual-recognition aptazyme beacon.

## Data Availability

Not applicable.
